# Prevalence and Abundance of Ixodid Ticks in Domestic Mammals in Villages at the Forest Fringes of the Western Ghats, India

**DOI:** 10.3390/ani15142005

**Published:** 2025-07-08

**Authors:** Hari Kishan Raju, Ayyanar Elango, Ranganathan Krishnamoorthi, Manju Rahi

**Affiliations:** 1 Climate Change, GIS and VBD Stratification/Mapping, ICMR-Vector Control Research Centre, Department of Health Research, Ministry of Health & Family Welfare, GOI, Medical Complex, Indira Nagar, Puducherry 605 006, India; krish.1267@gmail.com; 2 Division of Vector Biology and Control, ICMR-Vector Control Research Centre, Department of Health Research, Ministry of Health & Family Welfare, GOI, Medical Complex, Indira Nagar, Puducherry 605 006, India; elangoar@yahoo.co.in; 3 ICMR-Vector Control Research Centre, Department of Health Research, Ministry of Health & Family Welfare, GOI, Medical Complex, Indira Nagar, Puducherry 605 006, India; drmanjurahi@gmail.com

**Keywords:** tick surveillance, *Haemaphysalis spinigera*, *Ixodes ceylonensis*, Kyasanur Forest Disease, tick collection

## Abstract

Kyasanur Forest Disease (KFD), first reported in 1957 in the Shimoga district of Karnataka, India, has spread significantly over the past two decades, reaching both northern and southern states, with reports of monkey deaths. *Haemaphysalis* spp. ticks are the primary vectors, transmitting the disease to monkeys, humans, and other mammals. This study aimed to assess the prevalence, mean abundance, and mean intensity of Ixodidae ticks, including the KFD vector, in domestic animals across selected localities of the Western Ghats. A total of 2877 domestic animals were surveyed, revealing an overall tick prevalence of 44.91% (CI: 43.10–46.73), with sheep showing the highest prevalence at 47.92% (CI: 40.96–54.95). The most abundant tick species was *Rhipicephalus (Boophilus) microplus*, with a mean of 2.53 ± 0.66 ticks per host, which also represented the most proportionally dominant species, accounting for 39.63% of the total ticks collected. The highest mean intensity was recorded for *Haemaphysalis intermedia* (7.35 ± 2.03 ticks per infested animal). Regionally, *Rh. (Bo.) microplus* was found in 96.15% of buffaloes examined in Tamil Nadu, *Haemaphysalis bispinosa* in 85.19% of cattle in Maharashtra, and in 98.46% of goats in Goa. *Ha. intermedia* was common in 99.11% of sheep examined in Karnataka, while *Ha. bispinosa* was observed in 90.82% of goats in Kerala. The proportional representation of the KFD vector *Haemaphysalis spinigera* was 0.97%, with a mean intensity of 2.34 ± 0.04 ticks per infested animal and an overall mean abundance of 0.06 ± 0.01 ticks per host. Adult *Ha. spinigera* were recorded from cattle, buffaloes, sheep, goats, and dogs; however, no nymphs were detected. This study also reports the first documented occurrence of *Ixodes ceylonensis* in domestic animals. These findings suggest a notable presence of tick infestations in the region and emphasize the importance of continued surveillance and targeted control measures to better understand and manage potential KFD transmission risks in the Western Ghats.

## Simple Summary

Our study presents a comprehensive survey of tick populations infesting domestic mammals across the Western Ghats, a region with significant ecological diversity. This research includes a focus on tick species known to be involved in the transmission cycle of Kyasanur Forest Disease (KFD), a zoonotic disease that has expanded geographically in parts of India over recent decades. Through an extensive examination of 2877 domestic animals in selected localities of the Western Ghats, we report data on tick species prevalence, mean abundance, and mean intensity. Notably, we provide the first documented occurrence of *Ixodes ceylonensis* in domestic animals. The presence and distribution of medically important species such as *Haemaphysalis spinigera* are also discussed in the context of their potential vector roles. Our findings contribute to a better understanding of tick species ecology and host associations in this understudied region and provide a valuable baseline for future tick surveillance efforts in domestic animals.

## 1. Introduction

Ticks (Order: Ixodida) are obligate blood-feeding ectoparasites that infest a wide range of vertebrate hosts, including mammals, birds, and reptiles. They play a major role in veterinary and public health due to their capacity to transmit a wide array of pathogens such as viruses, bacteria, and protozoa. The Indian subcontinent harbors a rich diversity of tick species, particularly in ecologically varied regions like the Western Ghats, which feature multiple climatic zones and vegetation types conducive to tick survival and propagation [1,2].

The Western Ghats is known to support numerous Ixodid tick species, many of which infest domestic mammals such as cattle, buffaloes, goats, sheep, and dogs. Despite the ecological significance of this region, tick diversity and host associations in domestic animals remain understudied, particularly outside areas already known to be endemic for tick-borne diseases [3,4]. Understanding the composition, abundance, and prevalence of tick fauna in domestic animals is essential for anticipating the emergence and spread of tick-borne infections.

One such infection is Kyasanur Forest Disease (KFD), a tick-borne zoonotic illness caused by a flavivirus. KFD was first reported in 1957 in the Kyasanur Forest of the Shimoga district, Karnataka, and has since expanded into parts of Kerala, Goa, Maharashtra, and Tamil Nadu [5,6]. Retrospective studies show its geographic range has significantly widened over time [7]. Approximately 15 tick species have been implicated in KFD transmission cycles through virus detection in field-collected specimens, with *Haemaphysalis spinigera* identified as the principal vector [8,9]. Nymphs of *Ha. spinigera*, which are highly anthropophilic and abundant on the forest floor, are considered the most infective stage [4].

However, the detection of KFDV in ticks—particularly adults—is inconsistent. This may be due to transstadial but not transovarial transmission, where the virus is retained across molting stages (larva to nymph, and nymph to adult) but not passed from female ticks to their eggs [1,10,11]. This limits vertical transmission and emphasizes the importance of horizontal cycles involving vertebrate hosts. Additionally, adult ticks, especially males, often show reduced or no feeding activity, and females feed only once, reducing their opportunity to transmit the virus [11,12]. Adults may also have lower viral loads or reduced ecological overlap with competent amplifying hosts, or may die before completing transmission [12,13]. Although *Ha. spinigera* has been well-documented in some KFD-endemic parts of the Western Ghats, comprehensive studies on tick diversity and host interactions, particularly in forest-fringe domestic environments, remain limited [6,7,14].

This study aims to investigate the diversity, abundance, and host associations of Ixodid ticks in domestic animals across selected villages along the forest fringes of the Western Ghats. In doing so, we also report the occurrence of medically important species such as *Ha. spinigera*, providing baseline data to support future tick surveillance and control strategies in both KFD-affected and unaffected regions.

## 2. Materials and Methods

### 2.1. Study Area

**Figure 1:**
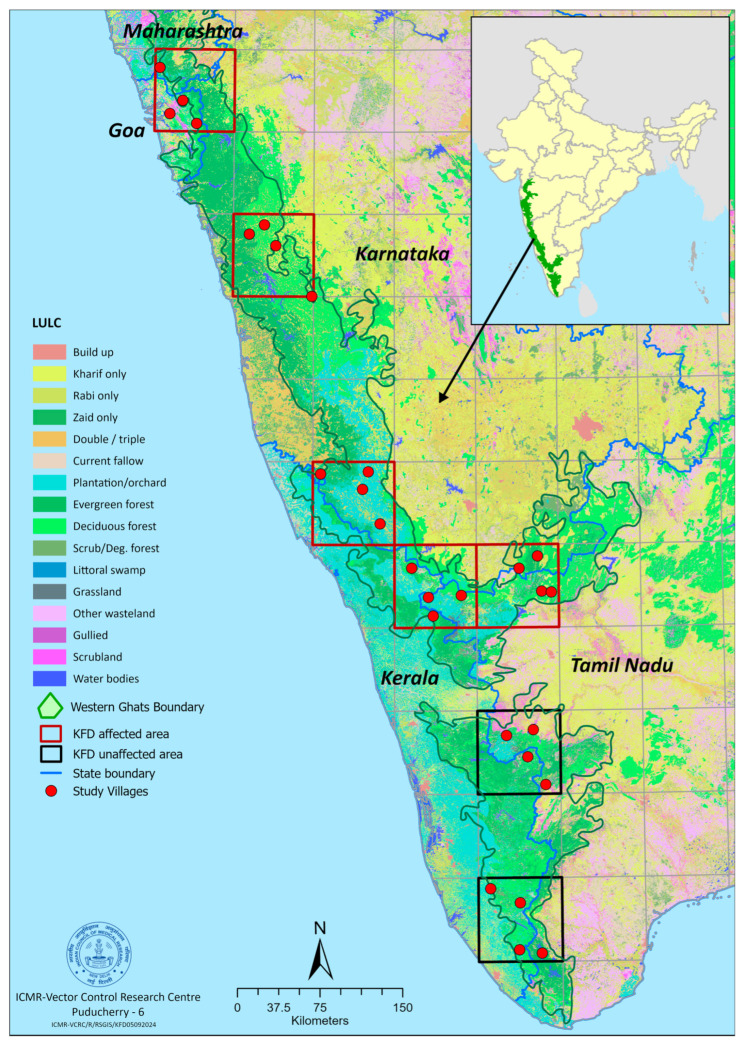
Western Ghats study area showing KFD-affected and -unaffected areas.

The Western Ghats, located along the west coast of India, covers an area of 160,000 km^2^, stretching over 1600 km across the states of Kerala, Tamil Nadu, Karnataka, Goa, Maharashtra, and Gujarat. The entire Western Ghats region was divided into grids of 75 × 75 square kilometers each. From the resulting 48 grids, seven grids were randomly selected for the study. In each selected grid, four villages situated along the forest fringes were randomly chosen for the tick survey (Figure 1).

Tick surveys were conducted on domestic animals in 28 villages across seven grids, representing five states: Goa, Karnataka, Kerala, Maharashtra, and Tamil Nadu. A total of 2877 domestic animals were examined for tick infestation. These included five host types: cattle (1884), buffaloes (191), sheep (192), goats (556), and dogs (54).

### 2.2. Adult and Nymph Collection

Animal ethical clearance was obtained from the Institutional Animal Ethics Committee (ICMR-VCRC/IAEC/2021-A/1). Tick collection was carried out between June and November 2022. Surveys were conducted in the morning hours between 7:00 a.m. and 9:00 a.m. The entire body surface of each animal was examined for ticks, with informed consent obtained from the animal owners.

Ticks were removed using fine-tipped tweezers held at a 45-degree angle to avoid damaging the specimens. Collected ticks were placed in micro-centrifuge tubes containing 80% ethanol. In each village, a maximum of 10 infested animals per host species (cattle, buffalo, goat, sheep, and dog) were sampled, or the collection was limited to two person-hours, whichever occurred first. For each animal, the presence or absence of ticks was recorded to estimate prevalence, mean intensity, and mean abundance. The estimated sample size was informed by an expected tick prevalence of approximately 45%, based on previous entomological surveys in similar ecological zones [14,15].

The following standard tick indices were calculated as described in previous studies.

Prevalence (%) was calculated as the number of infested animals divided by the total number of animals examined. To account for sampling variability and potential bias due to non-random host selection, confidence intervals (CIs) for prevalence were computed using the Wilson score method, which is considered appropriate for proportion data with small or moderate sample sizes [16,17]. All analyses were performed using the R version 4.1 (R Core Team, 2021), Binom package.

Mean Abundance was calculated as the total number of ticks collected divided by the total number of animals examined.

Mean Intensity was calculated as the total number of ticks collected divided by the number of infested animals. These metrics were used in accordance with the standardized parasitological definitions outlined by [16,18].

### 2.3. Tick Identification

The collected ticks were transported to the laboratory, and identification at species level was carried out under a stereo microscope using standard taxonomical keys [11,19,20] and Each sample was classified by species, life stage (adult, nymph, or larva), and sex where applicable. The number of individuals per species per village was recorded and pooled for further analysis [21].

### 2.4. Statistical Analysis

The Marascuilo Procedure was employed to compare differences in tick prevalence (i.e., proportions) across host species and states, using the StatsToDo software environment (https://www.statstodo.com/CombineMeansSDs.php (accessed on 10 March 2025), StatsToDo Trading Pty, Queensland, Australia).

An independent samples *t*-test was conducted to compare the abundance of tick species between KFD-affected and -unaffected areas. The classification of these areas was based on documented human cases and monkey deaths reported in the available literature [4,22] and by the National Centre for Vector Borne Diseases Control (NVBDCP). Statistical analyses were performed using SPSS (IBM SPSS Statistics for Windows, Version 25.0; IBM Corp., Armonk, NY, USA). A *p*-value of less than 0.05 was considered indicative of statistical significance.

## 3. Results

### 3.1. Tick Prevalence

A total of 18,409 ticks were collected from domestic animals across the Western Ghats. The overall tick prevalence among host animals was as follows: cattle (45.97%, CI: 43.73–48.22), buffaloes (34.55%, CI: 28.18–41.54), sheep (47.92%, CI: 40.96–54.95), goats (44.06%, CI: 39.99–48.22), and dogs (42.60%, CI: 30.33–55.84). The overall tick prevalence across all domestic animals and states was 44.91% (Table 1).

Tick prevalence by state was as follows: Goa (41.15% CI: 33.61–49.27), Karnataka (38.96%, CI: 36.66–43.54), Kerala (56.67%, CI: 52.49–61.49), Maharashtra (48.51%, CI: 40.94–56.14), and Tamil Nadu (45.20%, CI: 41.03–49.44).

A chi-square test revealed no significant difference in overall tick prevalence among the five states (χ^2^ = 2.724, df = 4, *p* = 0.605). However, statistically significant differences were observed for cattle (χ^2^ = 15.68, df = 4, *p* = 0.0035) and sheep (χ^2^ = 38.59, df = 2, *p* < 0.0001). No significant differences were found for goats (χ^2^ = 9.10, df = 4, *p* = 0.069), dogs (χ^2^ = 0.88, df = 2, *p* = 0.644), or buffaloes (χ^2^ = 3.46, df = 3, *p* = 0.326).

### 3.2. Tick Abundance in Domestic Animals

The overall tick abundance across domestic animals in the Western Ghats was 6.40 ± 0.70 ticks per animal. The most abundant tick species was *Rh. (Bo.) microplus* (2.53 ± 0.66), followed by *Ha. bispinosa* (1.75 ± 0.27). The KFD vector *Ha. spinigera* showed a relatively low abundance of 0.06 ± 0.01 ticks per animal (Table 2).

#### Tick Abundance in Domestic Animals Across KFD-Affected and -Unaffected Regions

Buffalo:
In KFD-affected regions, *Ha. bispinosa* showed the highest abundance in buffaloes (2.43), followed by *Rh. (Bo.) microplus* (0.24). In KFD-unaffected regions, *Rh. (Bo.) microplus* was most abundant (5.56), followed by *Rhipicephalus haemaphysaloides* (0.22) (Table S7).
Cattle:
In KFD-affected regions, *Rh. (Bo.) microplus* had the highest abundance in cattle (4.25), followed by *Ha. bispinosa* (1.68). In unaffected regions, *Rh. (Bo.) microplus* remained the most abundant (2.57), followed by *Rhipicephalus (Boophilus) annulatus* (1.54) (Table S8).
Dog:
In KFD-affected regions, the most abundant tick species in dogs was *Rhipicephalus sanguineus* (1.98), followed by *Ha. bispinosa* (1.30) (Table S9).
Goat:
In KFD-affected regions, *Ha. intermedia* exhibited the highest abundance in goats (4.05), followed by *Ha. bispinosa* (2.56). In unaffected regions, *Ha. bispinosa* was most abundant (3.86), followed by *Ha. intermedia* (1.47) (Table S10).
Sheep:
In KFD-affected regions, *Ha. intermedia* showed the highest abundance in sheep (10.72), followed by *Ha. bispinosa* (0.07). In KFD-unaffected regions, *Ha. intermedia* continued to dominate (9.09), followed by *Rh. haemaphysaloides* (1.02) (Table S11).

### 3.3. Mean Intensity

In the Western Ghats, *Ha. intermedia* exhibited the highest overall mean intensity (7.35 ± 2.03 ticks per infested animal), followed by *Rh. (Bo.) microplus* (5.74 ± 2.47). The KFD vector *Ha. spinigera* showed a mean intensity of 2.34 ± 0.04 ticks per infested animal (Table 2).

#### Mean Intensity by State and Host

Tamil Nadu:
The highest mean intensity was observed for *Rh. (Bo.) microplus* in buffaloes (5.56) and cattle (6.21), *Ha. bispinosa* in goats (6.51), and *Ha. intermedia* in sheep (8.89). *Ha. spinigera* infestation was reported only in cattle, with a mean intensity of 1.50; no other domestic animals in the region were infested by this species (Table S2).
Maharashtra:
The highest mean intensities were recorded for *Rh. sanguineus* in buffaloes (3.67) and *Ha. bispinosa* in cattle (3.76) and in dogs (3.25). *Ha. spinigera* was found only in cattle, with a mean intensity of 1.33 (Table S3).
Goa:
In this region, *Ha. bispinosa* exhibited the highest mean intensity in cattle (5.99) and goats (3.05), while *Rh. sanguineus* was predominant in dogs (4.07). The mean intensity of *Ha. spinigera* was 2.96 in cattle and 1.25 in dogs (Table S4).
Karnataka:
The highest mean intensities were observed in *Ha. bispinosa* in buffaloes (5.12), *Rh. (Bo.) annulatus* in cattle (6.47), *Amblyomma integrum* in dogs (2.00), and *Ha. intermedia* in goats (7.69) and sheep (8.99). *Ha. spinigera* was recorded with intensities of 1.67 in buffaloes, 2.25 in cattle, and 2.00 in goats (Table S5).
Kerala:
In cattle, *Rh. (Bo.) microplus* had the highest mean intensity (6.12), while *Ha. bispinosa* was most intense in goats (6.06), and *Rh. haemaphysaloides* in sheep (1.50). *Ha. spinigera* was detected in both cattle and goats with a mean intensity of 1.00 each (Table S6).

### 3.4. Proportional Representation of Tick Species

Overall, *Rh. (Bo.) microplus* exhibited the highest proportional representation, comprising 39.63% of all ticks collected across the Western Ghats, followed by *Ha. bispinosa* at 27.39%. The KFD vector *Ha. spinigera* accounted for only 0.97% of the total ticks collected (Table 2).

#### 3.4.1. Proportional Representation of Adult Tick Species Across Domestic Hosts in the Western Ghats

In Tamil Nadu, among adult ticks, *Rh. (Bo.) microplus* was predominant in buffaloes (92.86%) and cattle (75.03%), while *Ha. intermedia* was most common in goats (63.13%) and sheep (87.23%). *Ha. spinigera* had minimal presence, constituting 0.10% of adult ticks and was observed exclusively in cattle (Table S2).

In Maharashtra, *Ha. bispinosa* showed high adult representation in buffaloes (76.23%) and cattle (85.53%), whereas *Rh. sanguineus* dominated in dogs (60%). *Ha. spinigera* comprised 1.75% of adult ticks collected from cattle (Table S3).

In Goa, *Ha. bispinosa* accounted for 69.84% of adult ticks in cattle and 98.27% in goats, while *Rh. sanguineus* was dominant in dogs (57.01%). *Ha. spinigera* represented 11.81% of adult ticks in cattle and 4.67% in dogs (Table S4).

In Karnataka, *Ha. bispinosa* was most abundant in buffaloes (70.83%) and dogs (0.75%), *Rh. (Bo.) microplus* was predominant in cattle (58.57%), and *Ha. intermedia* was highly prevalent in goats (86.30%) and sheep (99.61%). *Ha. spinigera* was detected in buffaloes (1.04%), cattle (1.54%), and goats (0.32%). Rare species included *Nosomma monstrosum* (3.12% in buffaloes) and *Ixodes ceylonensis* (0.02% in cattle) (Table S5).

In Kerala, *Rh. (Bo.) microplus* was the dominant adult species in cattle (59.47%), *Ha. bispinosa* in goats (86.55%), and *Rh. haemaphysaloides* in sheep (50.00%). *Ha. spinigera* showed very low representation, comprising 0.03% in cattle and 0.14% in goats (Table S6).

#### 3.4.2. Proportional Representation of Tick Immature Stages Across Domestic Hosts in the Western Ghats

In Tamil Nadu, immature stages of *Rh. (Bo.) microplus* accounted for 100% of ticks from buffaloes and 99.66% from cattle. *Ha. bispinosa* represented 52.24% of immatures in goats, while *Ha. intermedia* comprised 100% in sheep (Table S2).

In Maharashtra, *Rh. (Bo.) microplus* immatures made up 66.67% in buffaloes, and *Ha. bispinosa* constituted 80.00% in cattle (Table S3).

In Goa, *Ha. bispinosa* dominated the immature stage collections, representing 80.00% in cattle and 100% in goats (Table S4).

In Karnataka, *Ha. bispinosa* was the most abundant immature tick, comprising 100% in buffaloes, 69.08% in cattle, and 93.71% in goats. *Am. integrum* accounted for 100% of immature ticks collected from dogs (Table S5).

In Kerala, *Rh. (Bo.) microplus* was the most common immature species in cattle (59.26%), while *Ha. bispinosa* made up 99.71% of immature ticks in goats (Table S6).

### 3.5. Comparative Statistical Analysis of Tick Species Abundance in KFD-Affected and -Unaffected Regions

A total of 18,409 ticks were analyzed across five domestic hosts (buffalo, cattle, goat, dog, and sheep) to evaluate the distribution and abundance of tick species in KFD-affected versus -unaffected regions. The analysis revealed distinct patterns of host-specific species dominance and significant differences in tick abundance across regions.

Buffalo
In KFD-affected regions, *Ha. bispinosa* was the most abundant species (2.43 ticks/host), while *Rh. (Bo.) microplus* dominated in unaffected regions (5.56 ticks/host; *p* < 0.01). Other species like *Nosomma monstrosum*, *Am. integrum*, and *Ha. spinigera* were exclusively observed in affected areas but in low abundance (Table S7).
Cattle
*Rh. (Bo.) microplus* was the most prevalent species in both zones, with significantly higher abundance in affected areas (4.25 vs. 2.57; *p* < 0.001). *Ha. bispinosa* was also significantly more abundant in affected regions (1.68 vs. 1.04; *p* < 0.001), whereas species like *Rh. annulatus* and *Am. integrum* were significantly more common in unaffected areas. Notably, the KFD vector *Ha. spinigera* was significantly more abundant in affected areas (0.11 vs. 0.01; *p* < 0.001) (Table S8).
Dog
Ticks were recorded only in dogs from KFD-affected regions, with *Rh. sanguineus* (1.98) and *Ha. bispinosa* (1.30) being dominant. *Ha. spinigera* was detected at low abundance (0.09), while no ticks were observed in dogs from unaffected areas, suggesting a localized risk associated with forest-proximal exposure (Table S9).
Goat
*Ha. intermedia* was significantly more abundant in KFD-affected areas (4.05 vs. 1.47; *p* < 0.001), while *Ha. bispinosa* was significantly more prevalent in unaffected zones (2.56 vs. 3.86; *p* < 0.001). Other species such as *Rh. haemaphysaloides* and *Rh. simus* were also more common in unaffected areas. *Ha. spinigera* showed very low presence in both zones (Table S10).
Sheep
*Ha. intermedia* overwhelmingly dominated in both affected and unaffected zones, with slightly higher abundance in affected areas (10.72 vs. 9.09; *p* < 0.01). Conversely, species like *Rh. haemaphysaloides*, *Rh. sanguineus*, *and Rh. annulatus* were significantly more prevalent in unaffected regions. The presence of *Ha. bispinosa* was low but slightly higher in KFD zones (Table S11).

## 4. Discussion

Kyasanur Forest Disease Virus (KFDV), a flavivirus, is transmitted to humans and monkeys primarily through the bite of an infected nymphal tick [2]. Currently, 14 tick species belonging to three genera in the Ixodidae family, and one species from the Argasidae family, have been implicated as KFDV vectors through virus isolation from unfed ticks collected in the Western Ghats region. Among these, 10 of the 15 *Haemaphysalis* species reported from the Western Ghats have been confirmed as KFDV vectors [1]. Notably, *Ha. spinigera* and *Haemaphysalis turturis* play a crucial role in virus transmission, and their presence in the region is closely monitored to understand the dynamics of KFDV spread, particularly to humans and non-human primates [23].

Domestic and wild mammals serve as important hosts for maintaining adult Ixodidae tick populations in forest fringes and deep forest regions. Multiple studies have explored the prevalence of Ixodid ticks in domestic and wild animals across the Western Ghats in Karnataka [24,25], Kerala [8,15,23,26,27,28] and Tamil Nadu [29,30,31]. In total, 23 Ixodidae species have been reported from the Western Ghats [8], highlighting the region’s rich biodiversity and its significance as a habitat for various tick species. In Karnataka, the primary KFD vector, *Ha. spinigera*, accounted for 2.7% of the ticks collected from cattle and 4.2% from buffaloes, while *Ha. turturis* accounted for 0.2% and 0.9%, respectively [25]. In Kerala, both *Ha. spinigera* and *Ha. turturis* have been reported from domestic animals [15,31].

The present study confirms the presence of *Ha. spinigera* in multiple domestic hosts, including cattle, buffaloes, goats, sheep, and dogs, although in low proportional representation. This finding contributes to our understanding of the host range of *Ha. spinigera*, though its role in domestic transmission remains uncertain. Conversely, *Ha. turturis* was not detected in domestic animals during this survey, which may be attributed to environmental and ecological factors such as restricted cattle movement, deforestation, and land-use changes that limit the distribution of *Ha. turturis* near human habitations.

Nymphs that detach from hosts molt into adults without entering a dormant phase. These adults remain dormant under leaf litter until the onset of the monsoon, after which they emerge and quest on vegetation in search of hosts. The monsoon (June to October) provides the high humidity necessary for egg development and favors the survival of newly emerged larvae [1]. Previous studies have reported peak infestation of adult *Ha. spinigera* on cattle during July and August [25]. Infested cattle, while grazing, may play a key role in enriching the larval *Ha. spinigera* population within the forest floor litter [24].

Other Ixodid tick species also show seasonal fluctuations, with monsoon-associated peaks in abundance. However, no seasonality was observed in the populations of *Ha. bispinosa* and *Rh. (Bo.) microplus*, which are the dominant species infesting cattle and buffaloes in Karnataka. These species have been reported throughout the year [25,31]. Similar non-seasonal patterns were also observed in the current study across Goa, Kerala, Maharashtra, and Tamil Nadu, although in Karnataka, *Rh. (Bo.) microplus* and *Ha. intermedia* were the predominant species.

In terms of species richness, 16 tick species have previously been reported in domestic animals from Karnataka [24], while the present study identified 14. In Kerala, 18 tick species have been documented from the Western Ghats region [15], with seven found in domestic mammals [27,31]. while the present study identified nine. Previous studies in Tamil Nadu reported nine species in domestic animals [29], compared to 12 species identified in the present study. Overall, this study documented 14 species in Karnataka, nine in Kerala, seven in Goa and Maharashtra, and 12 in Tamil Nadu. Differences in reported diversity across studies may be attributed to geographical variation, seasonality, host availability, and sampling intensity. Environmental factors such as deforestation and livestock grazing patterns also influence tick species abundance and distribution in forest fringe areas [32,33].

Notably, immature KFD vectors have not been reported on domestic animals in previous studies, and the present study in Karnataka supports this finding [1]. KFD vectors such as *Ha. spinigera* and *Ha. turturis* accounted for approximately 70% of the forest-floor tick population in the Western Ghats, and prior studies have confirmed their vector status through virus isolation from field-collected specimens [22]. Both the present study and previous studies have shown that there is no conclusive evidence on the specific host preferences of immature and adult stages of these vectors, as tick–host associations in the KFD-affected landscape remain poorly defined and are often inferred from limited field collections [13,23,28]. For effective control measures, further studies on the host preference of both immatures and adults of KFD vectors are needed. Based on the existing literature, small mammals and monkeys are likely hosts for the immature stages of the KFD vectors [22].

The occurrence of *Ixodes ceylonensis* on domestic animals is likely due to accidental host association. However, the identification of a female specimen from a domestic animal host in India is noteworthy, as it marks the first such report. Previously, it had only been recorded from wild animals and small mammals in forested areas, both in India and globally [34,35]. Further investigations are necessary to better understand the host range and ecological significance of this species.

## 5. Conclusions

This study provides important insights into the prevalence, mean abundance, mean intensity, and distribution of tick species on domestic animals along the Western Ghats of India, with particular attention to the presence of known KFD vectors such as *Ha. spinigera*. While domestic animals are not the primary hosts for the immature or adult stages of KFD vectors, they still serve as hosts for a variety of other tick species. The most prevalent tick species found on domestic animals was *Rh. (Bo.) microplus*, followed by *Ha. bispinosa*. These ticks were most commonly observed on cattle, buffaloes, goats, and sheep, with varying prevalence depending on the region.

Villages situated along the forest fringes exhibited a greater diversity of tick species, likely due to the ecological overlap between forest and domestic habitats. In contrast, villages deeper within the forest or farther from agricultural activities, with fewer small ruminants such as sheep and goats, showed reduced host availability; however, *Ha. intermedia* exhibited the highest abundance in these hosts when present.

Although *Ha. spinigera*, the primary vector of KFD, was detected in low prevalence and intensity on domestic animals, the epidemiological significance of its presence in these hosts remains unclear. Further investigation is required to determine whether domestic animals contribute meaningfully to the KFD transmission cycle.

Enhanced tick surveillance and species identification, especially near forest-adjacent villages, will be important for understanding vector distribution and potential disease risk.

## Figures and Tables

**Table 1:** Summary of tick prevalence in domestic hosts across the various Western Ghats states.

Name of the State	Cattle					Sheep					Goat					Dog					Buffalo					Total—No. of Host Screened	Total—No. of Host Infested	Total—Prevalence (%)	95 % Confidence Interval	
	No. of Host Screened	No. of Host Infested	Prevalence (%)	95 % Confidence Interval		No. of Host Screened	No. of Host Infested	Prevalence (%)	95 % Confidence Interval		No. of Host Screened	No. of Host Infested	Prevalence (%)	95 % Confidence Interval		No. of Host Screened	No. of Host Infested	Prevalence (%)	95 % Confidence Interval		No. of Host Screened	No. of Host Infested	Prevalence (%)	95 % Confidence Interval						
				Lower	Upper				Lower	Upper				Lower	Upper				Lower	Upper				Lower	Upper				Lower	Upper
Goa	148	61	41.21	33.61	49.27	0	0	0.0	0.00	0.00	38	13	34.21	21.21	50.11	23	12	52.17	32.96	70.76	0	0	0.00	0.00	0.00	209	86	41.15	34.70	47.92
Karnataka	774	310	40.05	36.66	43.54	146	52	35.62	28.31	43.66	188	77	40.96	34.18	48.1	8	2	25.0	7.15	59.07	111	37	33.33	25.25	42.53	1227	478	38.96	36.27	41.72
Kerala	461	263	57.05	52.49	61.49	2	1	50.0	9.45	90.55	167	93	55.69	48.11	63.01	0	0	0.0	0.00	0.00	0	0	0.0	0.00	0.00	630	357	56.67	52.77	60.48
Maharashtra	40	31	77.5	62.5	87.68	0	0	0.0	0.00	0.00	0	0	0.0	0.00	0.00	23	9	39.13	22.16	59.21	71	25	35.21	25.12	46.82	134	65	48.51	40.21	56.89
Tamil Nadu	461	201	43.6	39.15	48.16	44	39	88.64	76.02	95.05	163	62	38.04	30.94	45.68	0	0	0.0	0.00	0.00	9	4	44.44	18.88	73.33	677	306	45.2	41.49	48.96
Grand Total	1884	866	45.97	43.73	48.22	192	92	47.92	40.96	54.95	556	245	44.06	39.99	48.22	54	23	42.6	30.33	55.84	191	66	34.55	28.18	41.54	2877	1292	44.91	43.10	46.73

**Table 2:** Overall tick species composition, mean intensity, proportional representation of tick species (%), and mean abundance in Western Ghats of India.

Tick Species	Female				Male				Larva				Nymph				Total No. of Ticks	Total Mean Intensity ± SD *	Total %	Total Mean Abundance ± SD
	No. of Ticks	Mean Intensity	%	Mean Abundance	No. of Ticks	Mean Intensity	%	Mean Abundance	No. of Ticks	Mean Intensity	%	Mean Abundance	No. of Ticks	Mean Intensity	%	Mean Abundance				
*Am. integrum*	148	2.39	1.6	0.05	247	3.63	3.11	0.08	0	0.00	0.00	0.00	4	1.33	0.33	0.00	399	3 ± 1.02	2.17	0.14 ± 0.03
*Ha. bispinosa*	2391	5.13	25.92	0.83	1966	4.84	24.77	0.68	26	3.71	92.86	0.00	660	4.78	54.19	0.23	5043	4.96 ± 0.18	27.39	1.75 ± 0.27
*Ha. intermedia*	1218	5.14	13.2	0.42	2715	9.08	34.21	0.94	0	0.00	0.00	0.00	49	8.17	4.02	0.02	3982	7.35 ± 2.03	21.63	1.38 ± 0.47
*Ha. shimoga*	0	0.00	0.00	0.00	2	1.00	0.03	0.00	0	0.00	0.00	0.00	0	0.00	0.00	0.00	2	1 ± 0.00	0.01	0.00 ± 0.00
*Ha. spinigera*	88	2.32	0.95	0.03	90	2.37	1.13	0.03	0	0.00	0.00	0.00	0	0.00	0.00	0.00	178	2.34 ± 0.04	0.97	0.06 ± 0.01
*Hyalomma anatolicum*	7	2.33	0.08	0.00	25	2.08	0.31	0.00	0	0.00	0.00	0.00	0	0.00	0.00	0.00	32	2.13 ± 0.13	0.17	0.01 ± 0.00
*Hyalomma hussaini*	0	0.00	0.00	0.00	1	1.00	0.01	0.00	0	0.00	0.00	0.00	0	0.00	0.00	0.00	1	1 ± 0.00	0.01	0.00 ± 0.00
*Ixodes ceylonensis*	1	1.00	0.01	0.00	0	0.00	0.00	0.00	0	0.00	0.00	0.00	0	0.00	0.00	0.00	1	1 ± 0.00	0.01	0.00 ± 0.00
*Nosomma monstrosum*	7	2.33	0.08	0.00	8	4.00	0.10	0.00	0	0.00	0.00	0.00	0	0.00	0.00	0.00	15	3 ± 0.83	0.08	0.00 ± 0.00
*Rh (Bo.) annulatus*	654	6.61	7.09	0.23	293	3.15	3.69	0.10	0	0.00	0.00	0.00	42	3.00	3.45	0.01	989	4.8 ± 1.56	5.37	0.34 ± 0.10
*Rh (Bo.) microplus*	4460	7.05	48.34	1.55	2371	4.78	29.87	0.82	2	1.00	7.14	0.00	463	3.31	38.01	0.16	7296	5.74 ± 2.47	39.63	2.53 ± 0.66
*Rh. bursa*	31	3.1	0.34	0.01	29	2.64	0.37	0.01	0	0.00	0.00	0.00	0	0.00	0.00	0.00	60	2.86 ± 0.32	0.33	0.02 ± 0.01
*Rh. haemaphysaloides*	88	2.00	0.95	0.03	70	1.49	0.88	0.02	0	0.00	0.00	0.00	0	0.00	0.00	0.00	158	1.74 ± 0.21	0.86	0.05 ± 0.01
*Rh. sanguineus*	87	2.9	0.94	0.03	88	2.67	1.11	0.03	0	0.00	0.00	0.00	0	0.00	0.00	0.00	175	2.78 ± 0.12	0.95	0.06 ± 0.01
*Rh. simus*	46	3.07	0.5	0.01	32	2.00	0.4	0.01	0	0.00	0.00	0.00	0	0.00	0.00	0.00	78	2.52 ± 0.54	0.42	0.03 ± 0.01
Grand Total	9226	5.62	100	3.21	7937	5.21	100	2.76	28	3.11	100	0.009	1218	4.05	100	0.42	18409	5.3 ± 1.67	100	6.40 ± 0.70

* SD Standard deviation.

## Data Availability

Data will be available at reasonable request.

## Supplementary Materials

The following supporting information can be downloaded at: https://www.mdpi.com/article/10.3390/ani15142005/s1, Table S1: Total Number of Host Examined in KFD Affected and Unaffected Areas in the Western Ghats region; Table S2: Tick species Composition, Mean intensity and proportional representation of tick species (%) in Tamil Nadu regions of Western Ghats; Table S3: Tick species Composition, Mean intensity and proportional representation of tick species (%) in Maharashtra regions of Western Ghats; Table S4: Tick species Composition, Mean intensity and proportional representation of tick species (%) in Goa regions of Western Ghats; Table S5: Tick species Composition, Mean intensity and proportional representation of tick species (%) in Karnataka regions of Western Ghats; Table S6: Tick species Composition, Mean intensity and proportional representation of tick species (%) in Kerala regions of Western Ghats. Table S7: Comparative Statistical Analysis of Mean abundance and proportional representation of tick species in KFD Affected and KFD Unaffected areas across the Western Ghats in Host-Buffalo; Table S8: Comparative Statistical Analysis of Mean Abundance and proportional representation of tick species in KFD Affected and KFD Unaffected areas across the Western Ghats in Host-Cattle; Table S9: Comparative Statistical Analysis of Mean abundance and proportional representation of tick species in KFD Affected and KFD Unaffected areas across the Western Ghats in Host-Dog; Table S10: Comparative Statistical Analysis of Mean abundance and proportional representation of tick species in KFD Affected and KFD Unaffected areas across the Western Ghats in Host-Goat; Table S11: Comparative Statistical Analysis of Mean Abundance and proportional representation of tick species in KFD Affected and KFD Unaffected areas across the Western Ghats in Host-Sheep.

## Abbreviations

The following abbreviations are used in this manuscript:

KFD	Kyasanur Forest Disease
KFDV	Kyasanur Forest Disease Virus
NVBDCP	National Centre for Vector Borne Diseases Control
CI	Confidence Interval
SD	Standard Deviation
