# Supplementary material for: Prevalence and Abundance of Ixodid Ticks in Domestic Mammals in Villages at the Forest Fringes of the Western Ghats, India
Source: Animals (Basel). 2025 Jul 8;15(14):2005. doi: 10.3390/ani15142005 (PMC12291773; doi:10.3390/ani15142005)
Supplement: Supplementary file 1 [file animals-15-02005-s001.zip › animals-3637094-supplementary.pdf]

Table S1. Total Number of Host Examined in KFD Affected and Unaffected Areas in the Western Ghats region

State	Host Examined in KFD Affected Areas					Host Examined in KFD Unaffected Areas					Total
	**Cattle**	**Goat**	**Sheep**	**Dog**	**Buffalo**	**Cattle**	**Goat**	**Sheep**	**Dog**	**Buffalo**	
Goa	148	38	0	23	0	0	0	0	0	0	209
Karnataka	774	188	146	8	111	0	0	0	0	0	1227
Kerala	212	46	0	0	0	249	121	2	0	0	630
Maharashtra	40	0	0	23	71	0	0	0	0	0	134
Tamil Nadu	215	96	0	0	0	246	67	44	0	9	677
**Total**	**1389**	**368**	**146**	**54**	**182**	**495**	**188**	**46**	**0**	**9**	**2877**

Table S2. Tick species Composition, Mean intensity and proportional representation of tick species (%) in Tamil Nadu regions of Western Ghats

Host/species	Female		Male		Adult Ticks %	Nymph		Immature stages %	Total No. of ticks	Mean Intensity	Total %
	No. of ticks	Mean Intensity	No. of ticks	Mean Intensity		No. of ticks	Mean Intensity				
Buffalo											
Rh (Bo.) microplus	22	5.5	4	1.33	92.86	24	12	100	50	5.56	96.15
Rh. haemaphysaloides	0	0	2	2	7.14	0	0	0	2	2	3.85
Buffalo Total	22	5.5	6	1.5	100	24	12	100	52	5.2	100
Cattle											
Am. integrum	102	2.76	195	4.64	9.92	1	1	0.34	298	3.73	9.42
Ha. bispinosa	35	3.89	36	4.5	2.48	0	0	0	71	4.18	2.25
Ha. intermedia	39	3	62	4.43	3.53	0	0	0	101	3.74	3.19
Ha. spinigera	1	1	2	2	0.10	0	0	0	3	1.5	0.09
Hyalomma anatolicum	6	3	19	2.38	0.87	0	0	0	25	2.5	0.79
Hyalomma hussaini	0	0	1	1	0.03	0	0	0	1	1	0.03
Rh (Bo.) annulatus	54	2.57	40	2.67	3.28	0	0	0	94	2.61	2.97
Rh (Bo.) microplus	1292	7.69	857	5.39	75.03	297	4.43	99.66	2446	6.21	77.36
Rh. bursa	10	3.33	12	2.4	0.77	0	0	0	22	2.75	0.7
Rh. haemaphysaloides	29	2.64	19	1.9	1.67	0	0	0	48	2.29	1.52
Rh. sanguineus	0	0	2	1	0.07	0	0	0	2	1	0.06

Rh. simus	26	4.33	25	2.5	1.78	0	0	0	51	3.19	1.61
Cattle Total	1594	5.88	1270	4.62	100	298	4.38	100	3162	5.15	100
Goat											
Ha. bispinosa	135	6.43	188	7.52	34.92	35	3.89	52.24	358	6.51	36.09
Ha. intermedia	233	5.07	351	7.02	63.13	32	10.67	47.76	616	6.22	62.1
Rh (Bo.) microplus	3	1.5	1	1	0.43	0	0	0	4	1.33	0.4
Rh. haemaphysaloides	0	0	2	1	0.22	0	0	0	2	1	0.2
Rh. simus	7	1.4	5	1.25	0.76	0	0	0	12	1.33	1.21
Goat Total	378	5.11	547	6.67	100	67	5.58	100	992	5.9	100
Sheep											
Ha. intermedia	204	8.87	199	8.65	87.23	15	15	100	418	8.89	87.63
Hyalomma anatolicum	0	0	1	1	0.22	0	0	0	1	1	0.21
Rh. haemaphysaloides	31	2.58	13	1.3	9.52	0	0	0	44	2	9.22
Rh. sanguineus	8	1.33	6	1.5	3.03	0	0	0	14	1.4	2.94
Sheep Total	243	5.93	219	5.76	100	15	15	100	477	5.96	100
**Grand Total**	**2237**	**5.74**	**2042**	**5.12**	**100**	**404**	**4.87**	**100**	**4683**	**5.37**	**100**

Table S3. Tick species Composition, Mean intensity and proportional representation of tick species (%) in Maharashtra regions of Western Ghats

Host/species	Female		Male		Adult Ticks %	Larva		Nymph		Immature stages %	Total No. of ticks	Mean Intensity	Total %
	No. of ticks	Mean Intensity	No. of ticks	Mean Intensity		No. of ticks	Mean Intensity	No. of ticks	Mean Intensity				
Buffalo													
Am. integrum	8	1.6	9	3	13.93	0	0	0	0	0.00	17	2.13	13.6
Ha. bispinosa	53	3.79	40	3.64	76.23	0	0	1	1	33.33	94	3.62	75.2
Rh (Bo.) microplus	0	0	1	1	0.82	0	0	2	2	66.67	3	1.5	2.4
Rh. sanguineus	5	2.5	6	6	9.02	0	0	0	0	0.00	11	3.67	8.8
Buffalo Total	66	3.14	56	3.5	100	0	0	3	1.5	100	125	3.21	100
Cattle													
Am. integrum	0	0	3	1.5	1.31	0	0	0	0	0.00	3	1.5	1.23
Ha. bispinosa	155	4.7	40	2.5	85.53	2	2	10	2	80.00	207	3.76	85.19
Ha. spinigera	3	1.5	1	1	1.75	0	0	0	0	0.00	4	1.33	1.65
Rh (Bo.) annulatus	2	2	0	0	0.88	0	0	3	3	20.00	5	2.5	2.06
Rh (Bo.) microplus	15	3	8	2	10.09	0	0	0	0	0.00	23	2.56	9.47
Rh. haemaphysaloides	0	0	1	1	0.44	0	0	0	0	0.00	1	1	0.41
Cattle Total	175	4.27	53	2.21	100	2	2	13	2.17	100	243	3.38	100
Dog													
Ha. bispinosa	24	4	2	1	34.66	0	0	0	0	0.00	26	3.25	34.67
Rh (Bo.) microplus	4	2	0	0	5.33	0	0	0	0	0.00	4	2	5.33
Rh. sanguineus	19	2.71	26	3.71	60.00	0	0	0	0	0.00	45	3.21	60
Dog Total	47	3.13	28	3.11	100	0	0	0	0	0.00	75	3.13	100
**Grand Total**	**288**	**3.74**	**137**	**2.80**	**95.94**	**2**	**2**	**16**	**2**	**0.00**	**443**	**3.28**	**100**

Table S4. Tick species Composition, Mean intensity and proportional representation of tick species (%) in Goa regions of Western Ghats

Host/species	Female		Male		Adult Ticks %	Larva		Nymph		Immature Stages %	Total No. of ticks	Mean Intensity	Total %
	No. of ticks	Mean Intensity	No. of ticks	Mean Intensity		No. of ticks	Mean Intensity	No. of ticks	Mean Intensity				
Cattle													
Am. integrum	3	1.5	1	1	0.57	0	0	0	0	0.00	4	1.33	0.55
Ha. bispinosa	263	6.26	228	6.71	69.84	14	3.5	10	1.67	80.00	515	5.99	70.26
Ha. spinigera	38	2.71	45	3.21	11.81	0	0	0	0	0.00	83	2.96	11.32
Rh (Bo.) annulatus	0	0	1	1	0.14	0	0	0	0	0.00	1	1	0.14
Rh (Bo.) microplus	95	4.75	28	2.8	17.50	0	0	6	1.5	20.00	129	3.79	17.6
Rh. sanguineus	0	0	1	1	0.14	0	0	0	0	0	1	1	0.14
Cattle Total	399	5.12	304	4.98	100	14	3.5	16	1.6	100	733	4.79	100
Dog													
Ah. integrum	0	0	0	0	0.00	0	0	1	1	100	1	1	0.93
Ha. bispinosa	28	4	13	1.86	38.32	0	0	0	0	0	41	2.93	37.96
Ha. spinigera	4	1.33	1	1	4.67	0	0	0	0	0	5	1.25	4.63
Rh. sanguineus	37	5.29	24	3	57.01	0	0	0	0	0	61	4.07	56.48
Dog Total	69	4.06	38	2.38	100	0	0	1	1	100	108	3.18	100
Goat													
Am. integrum	1	1	0	0	1.72	0	0	0	0	0	1	1	1.54
Ha. bispinosa	45	4.09	12	1.71	98.27	0	0	7	2.33	100	64	3.05	98.46
Goat Total	46	3.83	12	1.71	100	0	0	7	2.33	100	65	2.95	100
**Grand Total**	**514**	**4.80**	**354**	**4.21**	**100**	**14**	**3.5**	**24**	**1.71**	**100**	**906**	**4.33**	**100**

Table S5. Tick species Composition, Mean intensity and proportional representation of tick species (%) in Karnataka regions of Western Ghats

**Host/species**	**Female**		**Male**		**Adult Ticks %**	**Larva**		**Nymph**		**Immature Stages %**	**Total No. of ticks**	**Mean Intensity**	**Total %**
	**No. of ticks**	**Mean Intensity**	**No. of ticks**	**Mean Intensity**		**No. of ticks**	**Mean Intensity**	**No. of ticks**	**Mean Intensity**				
Buffalo													
Am. integrum	29	2.42	28	2.33	11.87	0	0	0	0	0	57	2.38	11.68
Ha. bispinosa	206	6.24	134	4.62	70.83	0	0	8	1.33	100	348	5.12	71.31
Ha. spinigera	4	2	1	1	1.04	0	0	0	0	0	5	1.67	1.02
Hyalomma anatolicum	0	0	3	1.5	0.62	0	0	0	0	0	3	1.5	0.61
Nosomma monstrosum	7	2.33	8	4	3.12	0	0	0	0	0	3	3.07	0.61
Rh (Bo.) microplus	31	5.17	9	1.8	8.33	0	0	0	0	0	40	3.64	8.2
Rh. haemaphysaloides	2	1	2	2	0.83	0	0	0	0	0	4	1.33	0.82
Rh. sanguineus	9	3	7	3.5	3.33	0	0	0	0	0	16	3.2	3.28
Buffalo Total	288	4.72	192	3.56	100	0	0	8	1.33	100	488	4.03	100
Cattle													
Am. integrum	3	1	7	1.17	0.21	0	0	0	0	0	10	1.11	0.21
Ha. bispinosa	875	5.5	582	4.48	31.11	0	0	105	3	69.08	1562	4.82	32.31
Ha. intermedia	44	3.38	80	3.64	2.65	0	0	0	0	0	124	3.54	2.56
Ha. shimoga	0	0	2	1	0.04	0	0	0	0	0	2	1	0.04
Ha. spinigera	37	2.47	35	2.06	1.54	0	0	0	0	0	72	2.25	1.49
Ixodes ceylonensis	1	1	0	0	0.02	0	0	0	0	0	1	1	0.02
Rh (Bo.) annulatus	127	7.94	93	5.17	4.70	0	0	0	0	0	220	6.47	4.55
Rh (Bo.) microplus	1875	6.77	868	4.38	58.57	2	1	45	1.5	30.92	2790	5.5	57.7
Rh. bursa	21	3	17	2.83	0.81	0	0	0	0	0	38	2.92	0.79

Rh. haemaphysaloides	6	2	8	2	0.30	0	0	0	0	0	14	2	0.29
Rh. sanguineus	2	1	0	0	0.04	0	0	0	0	0	2	1	0.04
Cattle Total	2991	6.03	1692	4.2	100	2	1	150	2.31	100	4835	5.01	100
Dog													
Am. integrum	0	0	0	0	0	0	0	2	2	100	2	2	33
Ha. bispinosa	3	1	0	0	0.75	0	0	0	0	0	3	1	50
Rh. sanguineus	1	1	0	0	0.25	0	0	0	0	0	1	1	16.67
Dog Total	4	1	0	0	100	0	0	2	2	100	6	1.2	100
Goat													
Am. integrum	2	1	4	2	0.48	0	0	0	0	0	6	1.5	0.43
Ha. bispinosa	82	3.42	61	3.39	11.38	10	5	124	6.2	93.71	277	4.33	19.8
Ha. intermedia	363	5.58	721	9.49	86.30	0	0	0	0	0	1084	7.69	77.48
Ha. spinigera	0	0	4	2	0.32	0	0	0	0	0	4	2	0.29
Rh (Bo.) microplus	11	2.75	5	2.5	1.27	0	0	9	3	6.29	25	2.78	1.79
Rh. haemaphysaloides	0	0	1	1	0.08	0	0	0	0	0	1	1	0.07
Rh. sanguineus	0	0	2	1	0.16	0	0	0	0	0	2	1	0.14
Goat Total	458	4.82	798	7.75	100	10	5	133	5.78	100	1399	6.27	100
Sheep													
Ha. bispinosa	6	2	4	2	0.31	0	0	0	0	0	10	2	0.63
Ha. intermedia	298	4.38	1267	11.95	99.61	0	0	0	0	0	1565	8.99	99.11
Rh. haemaphysaloides	1	1	1	1	0.08	0	0	0	0	0	2	1	0.13
Rh. sanguineus	1	1	0	0	0	0	0	0	0	0	1	1	6
Rh. simus	1	1	0	0	0	0	0	0	0	0	1	1	0.06
Sheep Total	307	4.15	1272	11.67	100	0	0	0	0	0	1579	8.63	100
**Grand Total**	**4048**	**5.55**	**3954**	**5.91**	**100**	**12**	**3**	**293**	**3.08**	**0**	**8307**	**5.55**	**100**

Table S6. Tick species Composition, Mean intensity and proportional representation of tick species (%) in Kerala regions of Western Ghats.

Host/species	Female		Male		Adult ticks %	Nymph		Immature Stages %	Total No. of ticks	Mean Intensity	Total %
	No. of ticks	Mean Intensity	No. of ticks	Mean Intensity		No. of ticks	Mean Intensity				
Cattle											
Ha. bispinosa	131	3.05	350	6.03	16.81	15	1.88	11.11	496	4.55	16.55
Ha. intermedia	4	4	2	2	0.21	1	1	0.74	7	2.33	0.23
Ha. spinigera	0	0	1	1	0.03	0	0	0	1	1	0.03
Hyalomma anatolicum	1	1	2	2	0.10	0	0	0	3	1.5	0.1
Rh (Bo.) annulatus	471	7.72	158	2.72	21.98	39	3	28.89	668	5.06	22.29
Rh (Bo.) microplus	1112	7.67	590	5.22	59.47	80	2.42	59.26	1782	6.12	59.46
Rh. haemaphysaloides	12	1.33	16	1.45	0.98	0	0	0	28	1.4	0.93
Rh. sanguineus	5	5	5	2.5	0.35	0	0	0	10	3.33	0.33
Rh. simus	1	1	1	1	0.07	0	0	0	2	1	0.07
Cattle Total	1737	6.63	1125	4.57	100	135	2.45	100	2997	5.32	100
Goat											
Ha. bispinosa	349	6.12	275	4.74	86.55	345	7.67	99.71	969	6.06	90.82
Ha. intermedia	33	4.13	33	4.71	9.15	1	1	0.29	67	4.19	6.28
Ha. spinigera	1	1	0	0	0.14	0	0	0	1	1	0.09
Rh. haemaphysaloides	5	1	4	1	1.25	0	0	0	9	1	0.84
Rh. sanguineus	0	0	9	2.25	1.25	0	0	0	9	2.25	0.84
Rh. simus	11	5.5	1	1	1.66	0	0	0	12	4	1.12
Goat Total	399	5.47	322	4.35	100	346	7.52	100	1067	5.53	100
Sheep											
Ha. bispinosa	1	1	1	1	33.33	0	0	0	2	1	33.33
Rh (Bo.) annulatus	0	0	1	1	16.67	0	0	0	1	1	16.67
Rh. haemaphysaloides	2	2	1	1	50.00	0	0	0	3	1.5	50
Sheep Total	3	1.5	3	1	100	0	0	0	6	1.2	100
**Grand Total**	**2139**	**6.35**	**1450**	**4.49**	**100**	**481**	**4.76**	**0**	**4070**	**5.35**	**100**

Table S7. Comparative Statistical Analysis of Mean abundance and proportional representation of tick species in KFD Affected and KFD Unaffected areas across the Western Ghats in Host-Buffalo

**Species**	**KFD affected**			**KFD unaffected**					**Confidence interval**	
	**No. of Ticks**	**%**	**Mean abundance**	**No.of Ticks**	**%**	**Mean abundance**	**t-value**	**P-value**	**Lower**	**Upper**
Am. integrum	74	12.07	0.41	0	0.00	0.00	0.92	0.05	-0.46	1.28
Ha. bispinosa	442	72.10	2.43	0	0.00	0.00	1.34	0.01	-1.14	5.99
Ha. spinigera	5	0.82	0.03	0	0.00	0.00	0.31	0.53	-0.15	0.20
Hy. anatolicum	3	0.49	0.02	0	0.00	0.00	0.30	0.55	-0.09	0.13
Nosomma monstrosum	15	2.45	0.08	0	0.00	0.00	0.31	0.53	-0.44	0.60
Rh (Bo.)microplus	43	7.01	0.24	50	96.15	5.56	-6.92	0.00	-6.84	-3.80
Rh. haemaphysaloides	4	0.65	0.02	2	3.85	0.22	-2.20	0.00	-0.38	-0.02
Rh. sanguineus	27	4.40	0.15	0	0.00	0.00	-446.00	0.37	-0.51	0.80
**Grand Total**	**613**	**100.0**	**3.37**	**52**	**100.00**	**5.78**	**-1.22**	**0.02**	**-6.31**	**1.49**

Table S8. Comparative Statistical Analysis of Mean Abundance and proportional representation of tick species in KFD Affected and KFD Unaffected areas across the Western Ghats in Host-Cattle

Species	Kfd Affected			KFD unaffected					Confidence interval	
	No. of Ticks	%	Mean abundance	No.of Ticks	%	Mean abundance	t-value	P-value	Lower	Upper
Am. integrum	18	0.20	0.01	297	9.54	0.60	-10.16	0	-0.70	-0.47
Ha. bispinosa	2336	26.38	1.68	515	16.54	1.04	2.69	0	0.17	1.11
Ha. intermedia	124	1.40	0.09	108	3.47	0.22	-2.38	0	-0.24	-0.02
Ha. shimoga	2	0.02	0.00	0	0.00	0.00	0.84	0.09	0.00	0.01
Ha. spinigera	159	1.80	0.11	3	0.10	0.01	3.04	0	0.04	0.18
Hy. anatolicum	0	0.00	0.00	28	0.90	0.06	-4.54	0	-0.08	-0.03
Hy_hussaini	0	0.00	0.00	1	0.03	0.00	-1.68	0.001	0.00	0.00
Ixodes ceylonensis	1	0.00	0.00	0	0.00	0.00	NA	NA	NA	NA
Rh. annulatus	226	2.55	0.16	762	24.47	1.54	-11.13	0	-1.62	-1.13
Rh (Bo.) microplus	5899	66.62	4.25	1271	40.82	2.57	4.61	0	0.97	2.39
Rh. bursa	38	0.43	0.03	22	0.71	0.04	-0.72	0.15	-0.06	0.03
Rh. haemaphysaloides	47	0.53	0.03	44	1.41	0.09	-2.30	0	-0.10	-0.01
Rh. sanguineus	3	0.03	0.00	12	0.39	0.02	-1.97	0	-0.04	0.00
Rh. simus	2	0.02	0.00	51	1.64	0.10	-4.16	0	-0.15	-0.05
**Grand Total**	**8855**	**100.00**	**6.38**	**3114**	**100.00**	**6.29**	**0.20**	**0.002**	**-0.74**	**0.91**

Table S9. Comparative Statistical Analysis of Mean abundance and proportional representation of tick species in KFD Affected and KFD Unaffected areas across the Western Ghats in Host-Dog

**Species**	**KFD affected**			**KFD unaffected**					**Confidence interval**	
	**No. of Ticks**	**%**	**Mean abundance**	**No.of Ticks**	**%**	**Mean abundance**	**t-value**	**P-value**	**Lower**	**Upper**
Am. integrum	3	1.59	0.06	0	0.00	0	NA	NA	NA	NA
Ha. bispinosa	70	37.04	1.30	0	0.00	0	NA	NA	NA	NA
Ha. spinigera	5	2.65	0.09	0	0.00	0	NA	NA	NA	NA
Rh (Bo.) microplus	4	2.12	0.07	0	0.00	0	NA	NA	NA	NA
Rh. sanguineus	107	56.61	1.98	0	0.00	0	NA	NA	NA	NA
Rh. simus	0	0.00	0.00	0	0.00	0	NA	NA	NA	NA
**Grand Total**	**189**	**100.00**	**3.50**	**0**	**0.00**	**0**	**NA**	**NA**	**NA**	**NA**

Table S10. Comparative Statistical Analysis of Mean abundance and proportional representation of tick species in KFD Affected and KFD Unaffected areas across the Western Ghats in Host-Goat

**Species**	**KFD affected**			**KFD unaffected**					**Confidence interval**	
	**No. of Ticks**	**%**	**Mean abundance**	**No.of Ticks**	**%**	**Mean abundance**	**t-value**	**P-value**	**Lower**	**Upper**
Am. integrum	7	0.28	0.02	0	0.00	0.00	1.09	0.03	-0.02	0.05
Ha. bispinosa	942	37.98	2.56	726	69.61	3.86	-2.24	0.00	-2.45	-0.16
Ha. intermedia	1490	60.08	4.05	277	26.56	1.47	3.84	0.00	1.26	3.89
Ha. spinigera	4	0.16	0.01	1	0.10	0.01	0.44	0.38	-0.02	0.03
Rh (Bo.) microplus	29	1.17	0.08	0	0.00	0.00	1.33	0.01	-0.04	0.20
Rh. haemaphysaloides	2	0.08	0.01	10	0.96	0.05	-3.42	0.00	-0.08	-0.02
Rh. sanguineus	2	0.08	0.01	9	0.86	0.05	-1.75	0.00	-0.09	0.01
Rh. simus	4	0.16	0.01	20	1.92	0.11	-2.71	0.00	-0.17	-0.03
**Grand Total**	**2480**	**100.0**	**6.74**	**1043**	**100.0**	**5.55**	**1.46**	**0.00**	**-0.42**	**2.80**

Table S11. Comparative Statistical Analysis of Mean Abundance and proportional representation of tick species in KFD Affected and KFD Unaffected areas across the Western Ghats in Host-Sheep

**Species**	**KFD affected**			**KFD unaffected**					**Confidence interval**	
	**No. of Ticks**	**%**	**Mean abundance**	**No. of Ticks**	**%**	**Mean abundance**	**t-value**	**P-value**	**Lower**	**Upper**
Ha. bispinosa	10	0.63	0.07	2	0.41	0.04	0.29	0.56	-0.15	0.20
Ha. intermedia	1565	99.11	10.72	418	86.54	9.09	0.71	0.01	-2.93	6.20
Hy. anatolicum	0	0.00	0.00	1	0.21	0.02	-1.79	0.00	-0.05	0.00
Rh. annulatus	0	0.00	0.00	1	0.21	0.02	-1.79	0.00	-0.05	0.00
Rh. haemaphysaloides	2	0.13	0.01	47	9.73	1.02	-6.32	0.00	-1.32	-0.69
Rh. sanguineus	1	0.06	0.01	14	2.90	0.30	-4.12	0.00	-0.44	-0.16
Rh. simus	1	0.06	0.01	0	0.00	0.00	0.56	0.26	-0.02	0.03
**Grand Total**	**1579**	**100.0**	**10.82**	**483**	**100.0**	**10.50**	**0.14**	**0.04**	**-4.29**	**4.92**
